# Telomere dysfunction in some pediatric congenital and growth-related diseases

**DOI:** 10.3389/fped.2023.1133102

**Published:** 2023-04-03

**Authors:** Bo Zheng, Jianhua Fu

**Affiliations:** Department of Pediatrics, Sheng Jing Hospital of China Medical University, Shenyang, China

**Keywords:** telomere, telomerase (TERT), pediatric diseases, infants, aging

## Abstract

Telomere wear and dysfunction may lead to aging-related diseases. Moreover, increasing evidence show that the occurrence, development, and prognosis of some pediatric diseases are also related to telomere dysfunction. In this review, we systematically analyzed the relationship between telomere biology and some pediatric congenital and growth-related diseases and proposed new theoretical basis and therapeutic targets for the treatment of these diseases.

## 1. Introduction

Telomeres are part of the genome at the end of a linear chromosome. The telomeric DNA of vertebrates is composed of TTAGGG repeats, which bind to a group of proteins that regulate their biological functions and protect them from being recognized as DNA damage. In DNA replication, the wear of the telomere length (TL) accompanies each division; hence, the telomere length gradually shortens with age. Further evidence shows that the TL wear caused by childhood stress and other factors is a risk factor for diseases in adulthood and even in later life (1). Lin et al. (2) proved that the early telomere wear in infants has a certain impact on their long-term growth. Consistent with this, TL abrasion is closely related to the development of the nervous system within 2 years after birth (3, 4). Increasing evidence show that telomere attrition is related to children's growth and pediatric diseases. Thus, we focus on the latest progress in understanding the molecular properties of telomere biology in some pediatric diseases.

## 2. Telomere dysfunction in preterm infants

**Figure 1:**
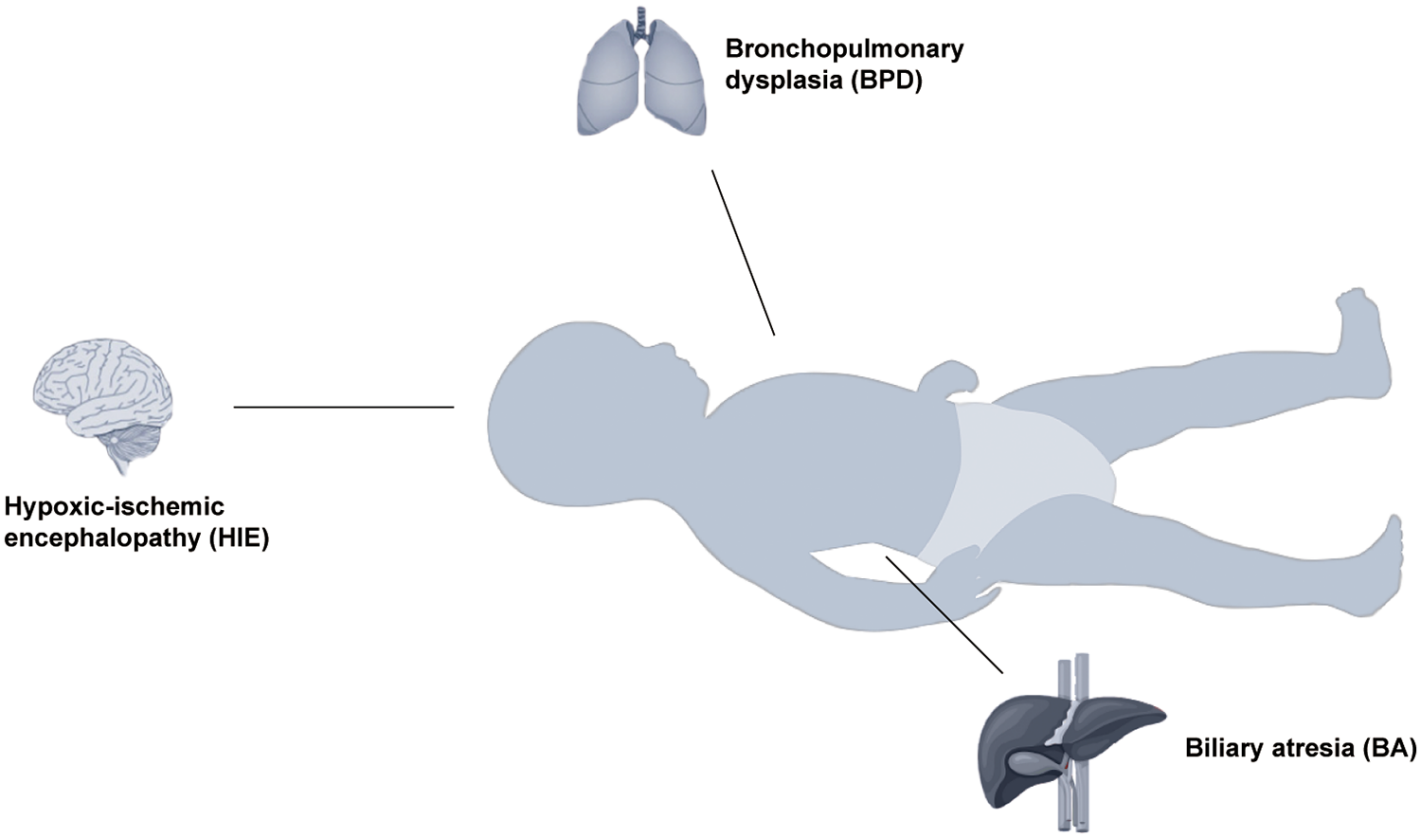
Evidence that telomere dysfunction causes some diseases in infants. For newborns, bronchopulmonary dysplasia is mainly associated with the respiratory system, hypoxic-ischemic encephalopathy with the nervous system, and biliary atresia with the digestive system.

Approximately, 15 million children are born preterm (at 37 weeks gestational age) yearly worldwide. Although the mortality rate of premature infants is decreasing with the continuous improvement of rescue technology in the neonatal intensive care unit, more than one million young people subsequently die of premature birth-related diseases each year. The degree of premature delivery is also closely related to the morbidity and mortality of surviving infants. Therefore, more research has been conducted on telomere dysfunction in premature infants (Figure 1 and Table 1); however, to date, the regulatory mechanism of premature infants is still unclear (8, 51–53). However, in detecting the TL of premature infants at birth, studies showed that the TL of premature infants decreased with the increase in gestational age, which was more obvious in premature infants less than 32 weeks of age (54, 55). Casavant et al. (56) demonstrated in a recent study that the mean absolute length of telomeres in the peripheral blood of premature infants is greater than that of adults. The TL of males gradually becomes shorter than that of females with age. They also studied the effects of pain stimuli exposure, feeding methods, and nervous system development on the TL of premature infants. The results show that these factors have no significant effect on the absolute length of telomeres. In contrast to this, some studies showed that exposure to adverse factors (such as pain and stress) during childhood is positively related with telomere shortening, and these factors can also affect their long-term mental health (57–59).

**Table 1:** List of telomere dysfunction-associated pediatric diseases and supporting evidence.

Disease	Telomere dysfunction	Telomere cell type		Animal model	Specific impact on disease	Refs.
Preterm Infants						
Biliary atresia (BA)	telomere attrition	human liver cells		no	shorter TL— severe liver cirrhosis	(5–7)
Bronchopulmonary dysplasia (BPD)	telomere attrition	cells from human saliva		no	shorter TL — lower FEF_25−75_ in extremely preterm infants	(8)
		human peripheral blood leukocytes		no	shorter TL— higher serum YKL-40	(9)
					shorter TL in early life of BPD children—severe outcome in adulthood	
Hypoxic-ischemic encephalopathy (HIE)	telomere attrition	nerve cells of central nervous system in rats		rat	TERT inhibition decreased the expression ratio of Bcl-2/Bax	(10, 11)
					the activated TERT has a protective effect in the hypoxic-ischemic neural model of rats	
Endocrine diseases						
Obesity	telomere attrition	human peripheral blood leukocytes		no	telomere attrition rate was greater	(12, 13)
			cross-sectional study	no	shorter TL— obesity—only in males	(14)
			cohort study	no	shorter TL— obesity—only in females	(15)
		human peripheral blood leukocytes		no	no association between TL and childhood obesity	(16, 17)
		human peripheral blood leukocytes		no­­	TL — positive correlation with SFA and DHA; negative correlation with AA/DHA ratio	(18)
Diabetes	telomere attrition	human peripheral blood leukocytes		no	shorter TL — T1D children	(19)
		human peripheral blood leukocytes		no	TL — all-cause mortality in T1D children	(20)
		human peripheral blood leukocytes		no	TL — negatively correlated with BMI-SDS in T1D children;	(21)
		human peripheral blood leukocytes		no	shorter TL — time of onset and course of disease; TL — negatively correlated with HbA1c	(22)
Hereditary diseases						
Trisomy 21 syndrome	longer telomeres		Data set interest generation analysis	no	trisomy 21 patients are born with longer telomeres; Higher telomere decay rate	(23)
Dyskeratosis congenita (DC)	TERT/TERC mutation	human peripheral blood leukocytes		no	TERC RNA levels were decreased in DKC1 —mutant cells	(24)
					mutated genes affecting TL: 1. Telomerase-associated components (TERC, TERT, DKC1, NOP10, NHP2, TCAB1, NAF1, PARN); 2. Shelterin proteins (TIN2, TPP1); 3. Regulators of TL (RTEL1, CTC1, STN1)	
		human/mice peripheral blood leukocytes		mice		(25–28)
Duchenne muscular dystrophy (DMD)	telomere attrition	mice muscle cells		mice	lack of TERC — severe muscular dystrophy	(29)
		diaphragm muscles of mice		mice	exercise factors can accelerate the telomere attrition	(30)
		cardiomyocytes		mice	TL—mechanosensitive in DMD	(31)
Cancers						
Neuroblastoma (NB)	TERT overexpression	human tumor pathological tissues and cells		no	TERT overexpression—Neuroblastoma	(32)
		neuroblastoma cell lines LAN-6, GI-ME-N, SK-N-FI		no	Shorter TL—Neuroblastoma cells with telomerase activated	(33)
Leukemia	High/Low TERT activity	human peripheral blood mononuclear cells		no	High TERT activity—ALL	(34)
		human bone marrow cells		no	Positive correlation between telomerase activity and CDKN2B	(35)
		human bone marrow cells		no	low TERT activity—AML	(36, 37)
Brain Tumors	High/Low TERT activity	human tumor pathological tissues		no	TERT activity—level of differentiation (low TERT activity—PLGGs; High TERT activity—PHGGs)	(38–41)
Other system diseases						
Cardiovascular system	telomere attrition	human peripheral blood leukocytes		no	shorter TL—IMT in later childhood	(42)
		human peripheral blood leukocytes		no	shorter TL—vascular elasticity but not thickness (only in adults but not in children)	(43)
Respiratory system	telomere attrition	fresh human frozen plasma		no	shorter TL—STRA and CCL11 expression in children	(44)
		human peripheral blood leukocytes		no	air pollution—shorter TL in children with asthma	(45)
Digestive system	telomere attrition	human colon tissue biopsies human peripheral blood lymphocytes and granulocytes		mice no	shorter TL—activation of ATM/YAP1— related to IBD shorter TL—not related to IBD	(46–48)
Urinary system	telomere attrition/TERT activity	human kidney pathological tissues		no	shorter TL—complications after renal transplantation in children	(49)
		human peripheral blood leukocytes		no	High TERT activity—in children with renal transplantation	(50)

In this table, we present the evidence, specific effects of telomere dysfunction and various diseases, and cell line models and/or animal pathological models.

### 2.1. Congenital biliary atresia (BA)

Congenital BA is the blockage of the intrahepatic and extrahepatic bile conduits, which can prompt cholestatic cirrhosis, and ultimately liver failure. It is one of the indications for liver transplantation in children and one of the most common digestive diseases in neonatal surgery. Patients with BA with hindered bile stream have determined jaundice, earth-like stool, hepatomegaly, and/or splenomegaly. If timely and reasonable intervention is not given in the early stage of the disease, most children may die at 2 years of age, with the cause of death mostly chronic liver disease (severe liver fibrosis, biliary cirrhosis, liver failure, etc.) (60). Hartley et al. (61) reported that the relative length of short telomeres in the peripheral blood of children was associated with the incidence of BA (adjusted for age and sex using logistic regression analysis). Their data also showed that the TL of patients with BA negatively correlated with the degree of liver cirrhosis. TL can also be used as one of the important indicators to evaluate the progress of BA in children (such as liver fibrosis) and is an important reference value for the timing of liver transplantation (5–7).

### 2.2. Bronchopulmonary dysplasia (BPD)

BPD is a chronic lung infection that causes constant respiratory misery. It is one of the most well-known serious diseases that influence preterm infants, particularly extremely preterm infants (Babies born before 28 weeks of pregnancy). In the long run, prolonged mechanical ventilation or oxygen inhalation may lead to stagnation of lung development, decline of vital capacity, emphysema, pulmonary hypertension, and even neurological and cognitive dysfunction. Nordlund et al. (62) suggested that BPD may damage the lung diffusion capacity of an infant. Airway hyperresponsiveness is more serious in premature infants than in full-term children with asthma at the same age. In a large-scale survey of young people with early pregnancy, Hadchouel et al. (8) showed that a shorter salivary TL was related with hindered lung capability. Previous studies also suggested that TL remained to be associated with forced expiratory flow at 25%–75% of the vital capacity in preterm adolescents, after adjusting for potential confounders by multiple linear regression. Henckel et al. (9) suggested that in children with early BPD, chitinase-3-like protein 1 and relative TL can be used as biomarkers for long-term lung development outcomes.

### 2.3. Hypoxic-ischemic encephalopathy (HIE)

Neonatal HIE is a severe disease caused by hypoxia in perinatal babies. Its normal cause is the fetal pain in the womb caused by different sources, such as the umbilical cord on the neck and abnormal amniotic fluid. It is very common in children who have experienced serious and life-threatening diseases (hypoxia, neonatal asphyxia) during or after childbirth, and occasionally other diseases that endanger the brain. This ordinarily happens in full-term babies, yet it can likewise happen in untimely newborn children. It has been reported that its incidence rate in live births was 1.5 ‰ (63). A total of 25% of neonates with HIE have severe neurological complications sooner or later (64).Telomerase reverse transcriptase (TERT) encodes rate-limiting catalytic subunits and key regulators of telomerase activity(65). Some studies reported that TERT is a protective molecule against hypoxic-ischemic brain damage. This theory has been verified in HIE models of neonatal rats (10, 11). Li et al. (66) believed that TERT can promote cell proliferation, increase the migration and separation of sensory cells, and then reduce the apoptosis of neurons after hypoxia-ischemia. This theory has been fully confirmed in *in vitro* experiments. Moreover, the overexpression of TERT can activate myelination in the brain of newborn rodents. In addition, TERT overexpression reduced learning disabilities, memory ability, and neural function after hypoxic-ischemic brain injury. They also conducted in-depth research on the neuroprotective mechanism of TERT and found that the sonic hedgehog/glioma-associated oncogene 1 signaling pathway may play an important and positive role.

## 3. Telomere dysfunction in nutritional and endocrine diseases in children

Telomere wear may be closely related to some endocrine diseases (such as diabetes) and may increase the early mortality of patients with these diseases. In some economically underdeveloped areas, children are more likely to experience various environmental stress factors, leading to malnutrition, stunting, and even fatality. Increasing evidence shows that telomere dysfunction is related to the nutritional status of and metabolic diseases in children.

### 3.1. Obesity in children

The incidence rate of childhood obesity is rising rapidly, as well as its related comorbidities, including obstructive sleep apnea syndrome, psychological problems, cardiovascular diseases, type 2 diabetes, and cancer. They seriously threaten the short- and long-term health of children. The telomerase plays a crucial role in human embryonic development. Telomerase action can result in a uniform TL in cells of different tissues, as well as differentiated cells (67–69). The shortening rates of telomeres are high during the first three years of life (approximately 250 bp/year) and reach a stable phase at 4 years of age. The loss rate of telomeres is stable or may decrease (approximately 50 bp/year) from the age of 4 years to youth and old age (approximately 50 bp/year) (70–74). In children with obesity, the wear rate of telomeres is increased (12, 13, 15). Different scholars hold different views on whether obesity is related to TL. Some research showed that there was a clear correlation between childhood obesity and shorter telomeres (14, 75, 76). In contrast, others reported that there was no clear correlation between obesity and TL (16, 17). Liu et al. (18) reported that in preschool children with obesity, leukocyte TL shortening negatively correlated with the body mass index. They also reported that the TL of peripheral blood lymphocytes negatively correlated with arachidonic acid (AA)/ docosahexaenoic acid (DHA) ratio but positively correlated with saturated fatty acid and DHA levels, respectively. In any case, no affiliation was found between erythrocyte DHA levels and the methylation of the TERT promoter. One study likewise focused on the effect of the sex distinctions of children with obesity on TL. Other relevant studies also indicated that only the shortening of TL in males was found to be associated with the incidence of obesity; however, this association was not obvious in females (14).

### 3.2. Diabetes in children

Diabetes in children is an endocrine and metabolic illness caused by the lack of insulin emission. This mainly results in carbohydrate, protein, and fat metabolism disorders, giving rise to fasting, postprandial hyperglycemia, and urine sugar. Type 1 diabetes (T1D) is common in young people and often occurs during childhood. Its main disease feature is the absolute lack of insulin, and it is a chronic immune system disease (77, 78). Previous studies showed that the TL of children with T1D was significantly shorter than that of healthy children. In-depth follow-up studies reported that shorter telomeres were associated with all-cause mortality (19, 20). Tesovnik et al. (21) reported that the average TL of young people with T1D was adversely connected with the body mass index standard deviation score, and that serum vitamin D levels were not associated with TL in these individuals. In another study by Tesovnik et al. (22), they reported that a shorter telomere indicates an earlier and longer onset and duration of T1D; however, TL negatively correlated with glycated hemoglobin A1c. In the study of Törn et al. (79), after a comprehensive sequencing test of the gene level of thousands of children with T1D, results showed that the age of onset negatively correlated with TL, and that children with HLA-DR4/4 or HLA-DR4/X phenotypes had longer telomeres compared with children with HLA-DR3/3 or HLA-DR/9 genes.

## 4. Telomere dysfunction in hereditary diseases of children

### 4.1. Trisomy 21 syndrome

Trisomy 21 syndrome, also known as Down syndrome (DS), is caused by chromosomal abnormalities (an additional chromosome 21). Moreover, 60% of Trisomy 21 pregnancies were miscarried in the early fetal period. Children with DS have clear mental retardation, exceptional faces, developmental obstacles, and multiple malformations. A comparative study of three different Indian trisomy 21 data sets by Bhattacharya et al. (23) indicated that the TL of children with trisomy 21 syndrome decreased by 58 bps/year. In contrast, the TL of normal children decreased by 38 bps/year. The telomere attenuation rate of children with trisomy 21 was evidently higher. However, Nakamura et al. (80) tested the peripheral lymphocytes (diploid, 10; trisomy 18, 10; trisomy 21, 11) of 31 live newborns. They observed that there was no measurable difference in TL among trisomy 18, trisomy 21, and diploid infants. In contrast, some studies showed that normal children have longer TL than children with trisomy 21 (81, 82). Thus, the above views on the divergence between TL and trisomy 21 must be further studied.

### 4.2. Dyskeratosis congenita (DC)

Dyskeratosis congenita (DC), a multisystem inherited condition, is characterized by skin symptoms and can lead to bone marrow regeneration disorder or tumor. The inheritance of DC can be X-linked latent, autosomal predominant, or autosomal passive. Practically, patients with X-linked latent DC were male. Hoyeraal–Hreidarsson disease, Revesz syndrome, or Coats plus disease is usually the first symptom of DC (83–86). Some sporadic, non-classical cases are usually related to DC-related reproductive biological gene mutations, which mainly manifested as aplastic anemia, familial myelodysplastic syndromes/acute myeloid leukemia (AML) carriers, etc (87–90).. Mutations in the genes encoding TERT, telomerase RNA component (TERC), and dyskerin pseudo uridine synthase 1 (DKC1) genes are all related to the pathogenesis of DC (91–93). Mitchell et al. (24) reported that the level of TERC RNA in the DKC1-mutant cells of patients with DC was reduced. In the past years, there has been increasing evidence that gene mutations that maintain telomere gene length may increase the incidence of DC by 70%. At present, 13 known genes have this effect (25–28). They include the genes encoding telomerase-associated components [TERC, TERT, DKC1, nucleolar protein 10, H/ACA ribonucleoprotein complex subunit 2, telomerase Cajal body protein 1, nuclear assembly factor 1, and poly(A)-specific ribonuclease], shelterin proteins (TERF1-interacting nuclear factor 2, tripeptidyl-peptidase 1), and other regulators of TL and replication (regulator of telomere elongation helicase 1, CTC1, STN1).

### 4.3. Duchenne muscular dystrophy (DMD)

DMD is an X-chromosome recessive disease, which mainly occurs in males. Approximately one in each 3,500 newborn infants have this disease. Patients usually experience progressive degeneration of skeletal muscles, which gradually leads to muscle atrophy and eventually loss of motor function. These manifestations generally occur during the early-school age. It is mainly characterized by the gradual replacement of necrotic skeletal muscle cells by fibrocytes and adipocytes. Gradually, patients lose their exercise ability before and after school age, leading to death during adulthood when respiratory muscles are involved in serious cases. This disease is caused by the lack of proteins connecting the cytoskeleton and extracellular matrix, resulting in serious destruction of the integrity of the skeletal muscle cell membrane. The main genetic factors of this disease have been studied; however, its main pathogenesis must be further investigated (94). Sacco et al. (29) reported that the TL of dystrophin-deficient (mdx) mouse muscle cells lacking telomerase RNA components was shortened, leading to extremely severe malnutrition, which gradually worsened with age. The severity of muscle atrophy parallels the decline of the regeneration ability of muscle stem cells. Vita et al. (30) showed that in an mdx mouse model, the TL of the diaphragm was significantly shortened, and exercise factors could accelerate the telomere wear rate. Chang et al. (31) showed that telomere shortening is progressive, contraction-dependent, and mechanically sensitive in cardiomyocytes of DMD mice.

## 5. Telomere dysfunction in pediatric cancers

The incidence of cancer in children ranges from 0.05 to 3 per 10,000, accounting for 1% of all types of cancer (95). Childhood cancer is mostly related to congenital and genetic factors, but not strongly related to environmental factors and poor lifestyle, which is different from adult cancer. The role of telomere dysfunction in the occurrence and development of childhood cancer has gained more attention in recent years.

### 5.1. Neuroblastoma (NB)

NB is the most common extracranial tumor in children and may originate from early nerve cells of the sympathetic nerve system. Currently, only a few human tumors are known to spontaneously degenerate from undifferentiated malignant tumors to completely benign tumors, including NB. Lindner et al. (32) reported that the increased expression of TERT was detected in most NBs. Telomerase activation occurs in parallel with telomere shortening in NB (33, 96). Ackermann et al. (97) reported that repeated genome rearrangement in the proximal 5 p15.33 chromosome region may be the reason for the overexpression of TERT in NB cells. In the biological behavior of NBs, telomerase activation and the maintenance of TL are very important and necessary links that provide relatively accurate molecular mechanisms that determine the clinical phenotype of the tumor (33).

### 5.2. Leukemia

Leukemia is a malignant proliferative disease of the human hematopoietic system, which seriously threaten children's lives. Among the types of childhood leukemia, acute lymphoblastic leukemia (ALL) and AML are the two most common, accounting for approximately 3% of childhood malignant tumors (98, 99). A study by Engelhardt et al. (34) used data from the telomerase activity of peripheral blood monocytes. Compared with healthy children, the telomerase activity of children with ALL was 10–20 times higher. Similarly, Hu et al. (35) reported that the methylation of the cyclin dependent kinase inhibitor 2B gene was closely related to the telomerase activity in childhood cancer, and that telomerase activation is one of the important monitoring indicators for childhood cancer. It is interesting to note that, unlike previous perceptions, the mutation of the gene with reduced TERT activity is an important risk factor for the disease in children with AML (36). The telomerase activity of bone marrow cells positively correlated with prognosis in adult patients with AML, but negatively correlated with prognosis in children with AML (37).

### 5.3. Brain tumors

Intracranial tumors are the most common tumors during childhood, which makes it a serious threat to the life and health of children. Its overall incidence in the population ranks second (only next to the incidence of leukemia). People of all ages may have this disease; however, its peak of occurrence is during 5–8 years of age. In recent years, some studies have shown the close relationship between the maintenance mechanism of TL and occurrence of intracranial tumors in children (100–102). In pediatric glioma, the level of differentiation is related to telomerase activity. Some studies suggested that according to the standards of the World Health organization, telomerase activity was significantly reduced in low-grade gliomas in children (38, 39). In contrast, telomerase activity is significantly enhanced in most children with high-grade glioma (40, 41). Telomerase activity was increased in primary medulloblastoma but did not change in secondary medulloblastoma (101, 103).

## 6. Pediatric diseases in other body systems

**Figure 2:**
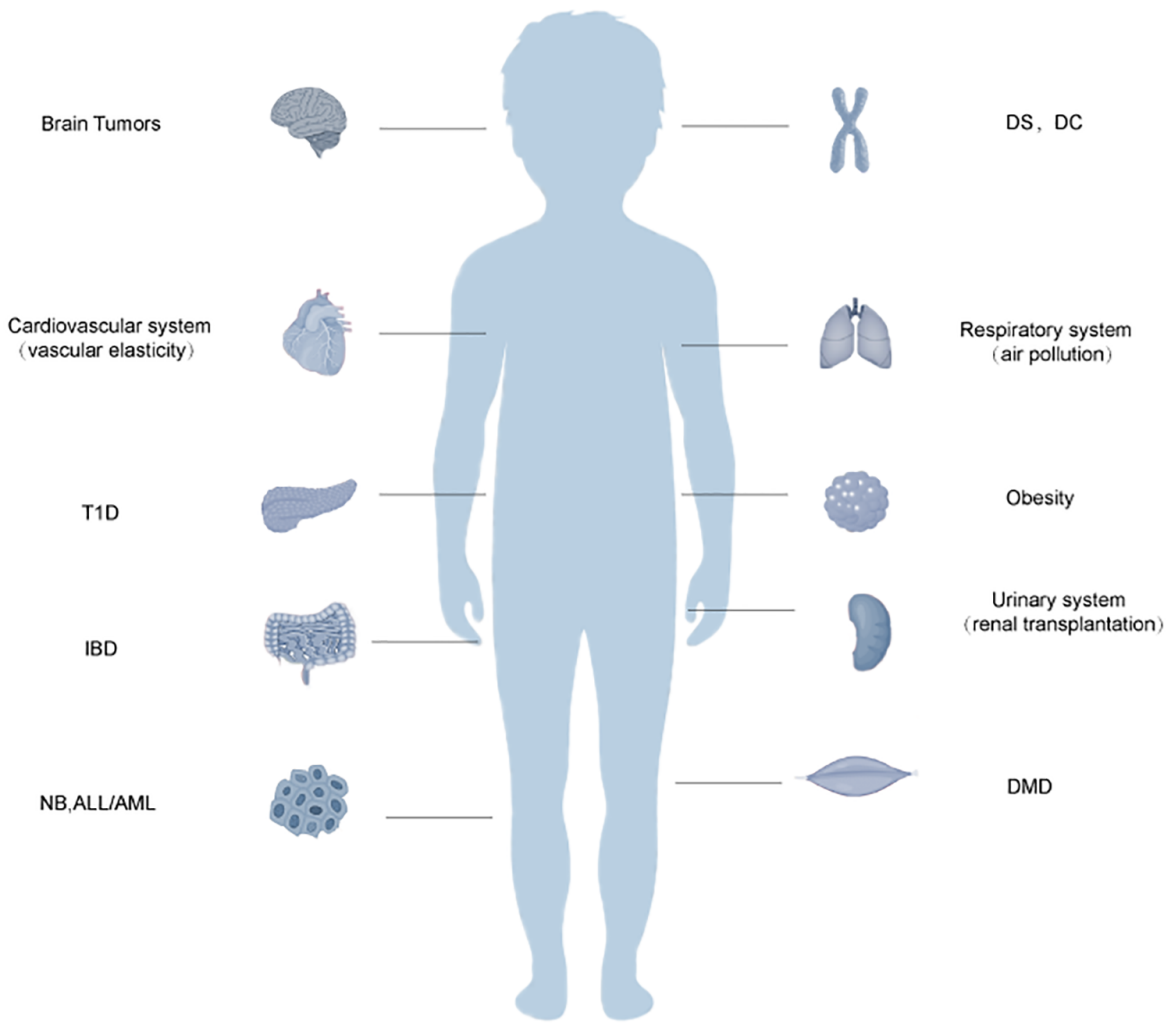
Schematic diagram of the relationship between telomere dysfunction and certain systemic disorders in older children. Acute lymphoblastic leukemia, ALL, acute myeloid leukemia; AML, dyskeratosis congenita; DC, Duchenne muscular dystrophy; DMD, Down syndrome; DS, inflammatory bowel disease; IBD, neuroblastoma; NB, type 1 diabetes, T1D. Telomere dysfunction may also be related to the body systems in children. It may be related to vascular elasticity in the cardiovascular system. In the respiratory system, it can enhance the sensitivity of the respiratory tract to air pollution. In the urinary system, it may be related to the success rate of renal transplantation.

Telomere dysfunction is rarely studied in the pediatric diseases of other body systems, such as the cardiovascular and urinary systems. Nevertheless, we included them in this review (Figure 2 and Table 1).

Carotid intima-media thickness (IMT) is a risk factor for cardiovascular events in adult patients. Skilton et al. (42) confirmed that a shorter TL in youth was related to carotid IMT at 8 years of age but not to early life risk factors (birth weight, gestational age, skewed weight gain at the beginning, asthma, and atopy). In contrast, Nguyen et al. (43) reported that in middle-aged adults, TL was related with vascular versatility but not thickness. The influence of the telomere on respiratory diseases in children mainly focuses on its effect on asthma. Barbe-Tuana et al. (44) suggested that telomere shortening is positively related to severe therapy-resistant asthma in children, and that telomere attrition was associated with increased plasma eotaxin-1 expression in children. Lee et al. (45) confirmed that in children with asthma, exposure to air pollution is related to a shorter TL and that telomere wear may be reduced by steroid intervention. Similarly, Isaevska et al. (104) reported that for pregnant mothers, air pollution factors were related to shorter telomeres at birth, which can increase the long-term incidence of asthma and other diseases in children. In the study of Chakravarti et al. (46) found that telomere dysfunction leads to activation of ATM/YAP1 pathway, which is related to the pathogenesis and severity of inflammatory bowel disease (IBD).However, some scholars also believe that no evidence has been found that TL is associated with IBD in children (47, 48).

For patients undergoing renal transplantation, TL can be used as one of the indicators of long-term renal function; moreover, the incidence of renal transplantation or long-term complications is positively correlated with shorter telomeres (49). In contrast to adult patients, in children undergoing renal transplantation, plasma TRF2 correlated with the creatinine level and epidermal growth factor. Notably, although there is no significant difference in TL between children receiving renal transplantation and healthy children, the telomerase activity of children undergoing renal transplantation was significantly increased (50).

## 7. Prospect of treatment

It can be inferred that most telomere dysfunction-related diseases are caused by TL wear; hence, considering how to enhance telomerase activity and reduce telomere wear may potentially be the main targets of treatment. There are currently two main views. One is to promote the increase in the endogenous expression of TERT. As an endogenous promoter of TERT, androgen has been widely used in the treatment of aplastic anemia (105). As a specific PAPD5 inhibitor, BCH001 can restore telomerase activity in patients with DC and reduce the TL wear of pluripotent stem cells (106). In a mouse model, TA-65 has been shown to delay telomere wear by increasing telomerase activity (107). Recently, as a therapeutic method, TERT was overexpressed in an adeno-associated virus vector and transfected into animal models. Significant achievements have been made in the treatment of some adult aging-related diseases such as Alzheimer's disease, aplastic anemia, and idiopathic pulmonary fibrosis (96, 108–110). However, these kinds of treatment have not been investigated in terms of the risk of tumor occurrence and pediatric application.

Telomere overexpression is common in most patients with pediatric cancers; hence, inhibiting telomerase activity may be a potential therapeutic target. The 1,3,4-Oxadiazole scaffold is a five-member heterocyclic ring, which can be used as a telomerase inhibitor in tumor therapy (111, 112). As a telomerase inhibitor, imetelstat also plays a significant role in the treatment of adult AML (113). It can competitively inhibit the binding of telomerase and the human telomerase RNA component sequence, thereby inhibiting telomerase activity. For myelofibrosis, myelodysplastic syndrome, and other hematological malignancies, the application of imetelstat has also achieved gratifying results (114). As discussed above, telomerase expression in adults and children with AML is different; thus, this treatment is not applicable to children.

Antisense oligodeoxynucleotides (ASO) are a new class of drugs, which have gained more attention. It was confirmed in a mouse model that telomere ASO can reduce the DNA abrasion caused by telomere abrasion (115, 116).

**Figure 3:**
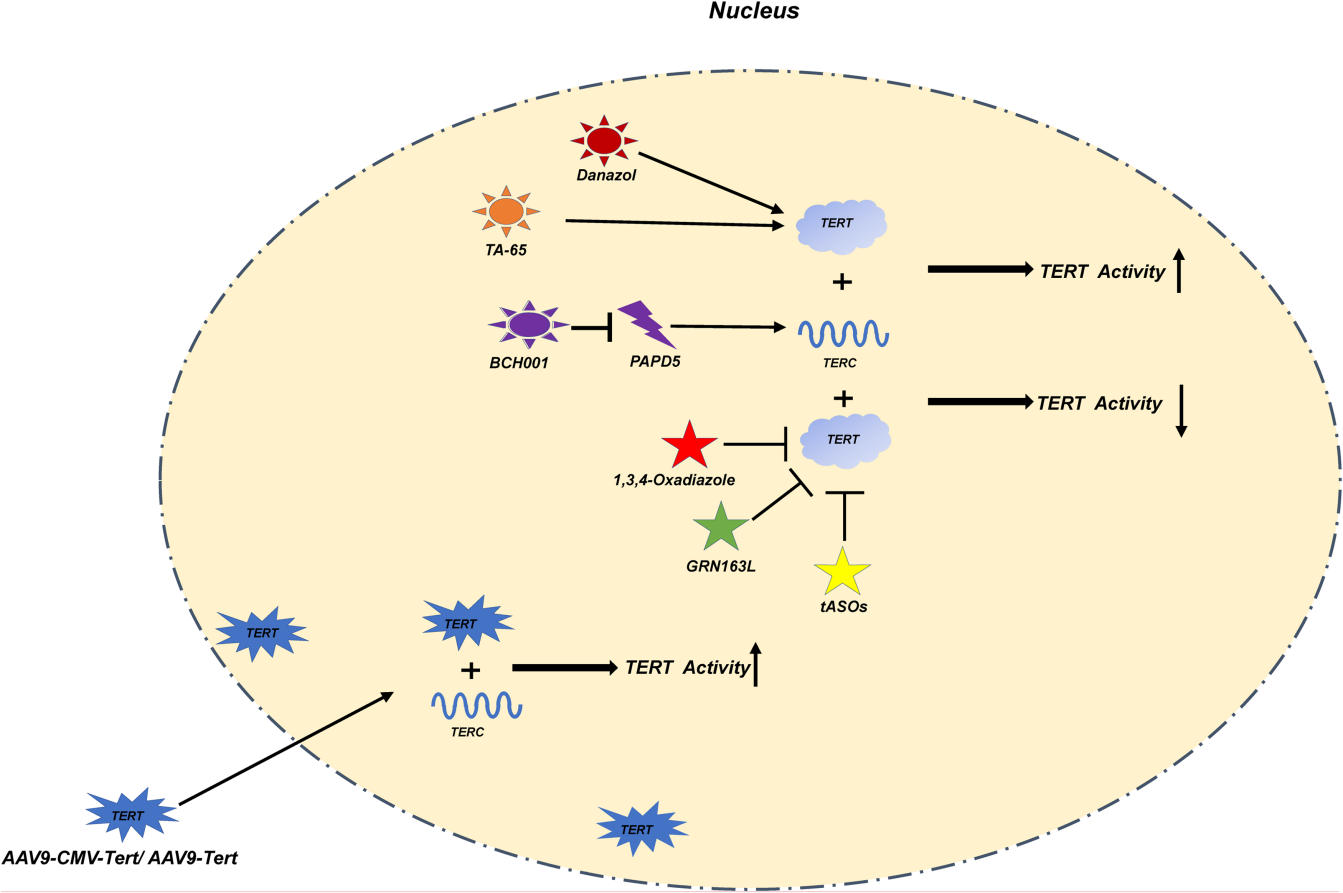
In this figure, we show various treatment methods and their mechanisms in detail. Danazol, TA-65 directly increase the activity of TERT; BCH001 reduces the consumption of TERC by inhibiting PAPD5 to improve the activity of TERT; The exogenous AAV9-CMV-Tert/AAV9-Tert enhances its activity by increasing the relative number of TERT in the nucleus; 1,3,4-Oxadiazole, GRN163L; ASOs inhibit the activity of TERT.

Most of the above treatments for telomeres are based on the treatment of diseases related to adult telomere dysfunction (Table 2 and Figure 3). Research on the treatment of telomere dysfunction-associated pediatric diseases is lacking. Therefore, both the change in telomerase activity and maintenance of TL may be potential targets for pediatric diseases.

**Table 2:** Prospect of treatment of telomere dysfunction-related diseases.

TERT activation	treatment type	Representative drugs	Drug action	Animal model	Disease	Refs.
(1) endogenous expression of TERT	Androgen	Danazol		no	AA	(105)
	specific PAPD5 inhibitor	BCH001	enhance the activity of TERT by reducing the consumption of TERC	no	DC	(106)
	A special ingredient is found in the root of astragalus	TA-65	an effective telomerase activator in human immune cells, and neonatal keratinocytes and fibroblasts	mice	Senescence	(107)
(2) Exogenous import TERT	gene therapy	AAV9-CMV-Tert	After overexpression of TERT *in vitro*, it was introduced into animal models through AAV	mice	AD	(108)
	gene therapy	AAV9-Tert	After overexpression of TERT *in vitro*, it was introduced into animal models through AAV	mice	osteoporosis, glucose intolerance, AA, IPF	(109)
	gene therapy	AAV9-Tert	After overexpression of TERT *in vitro*, it was introduced into animal models through AAV	mice	osteoporosis, neuromuscular coordination	(110)
TERT inhibitor						
	TERT inhibitor	1,3,4-Oxadiazole	Inhibiting the activity of TERT and then inhibiting the immortalization of tumor	no	broad-spectrum anticancer activity against different cell lines	(111, 112)
	Competitive inhibitors of TERT	GRN163L	It directly binds to TERC at the enzyme catalytic site to inhibit TERT activity	no	AML	(113, 114)
ASO	Short peptide chain	tASOs	Antisense strand RNA interference	no	DNA damage mediated by telomere attrition	(115, 116)

The therapeutic targets for diseases with telomere dysfunction mainly focus on the intervention of telomerase (TERT) activity. In this table, we present potential treatments for some diseases (not limited to pediatrics): TERT activation: (1) Endogenous expression of TERT: Danazol, BCH001 TA-65; (2) Exogenous import TERT: AAV9-CMV-Tert/ AAV9-Tert; TERT inhibitor: 1,3,4-Oxadiazole, GRN163L; ASOs (Antisense oligodeoxynucleotides): Antisense strand RNA interference.

## 8. Conclusions

**Figure 4:**
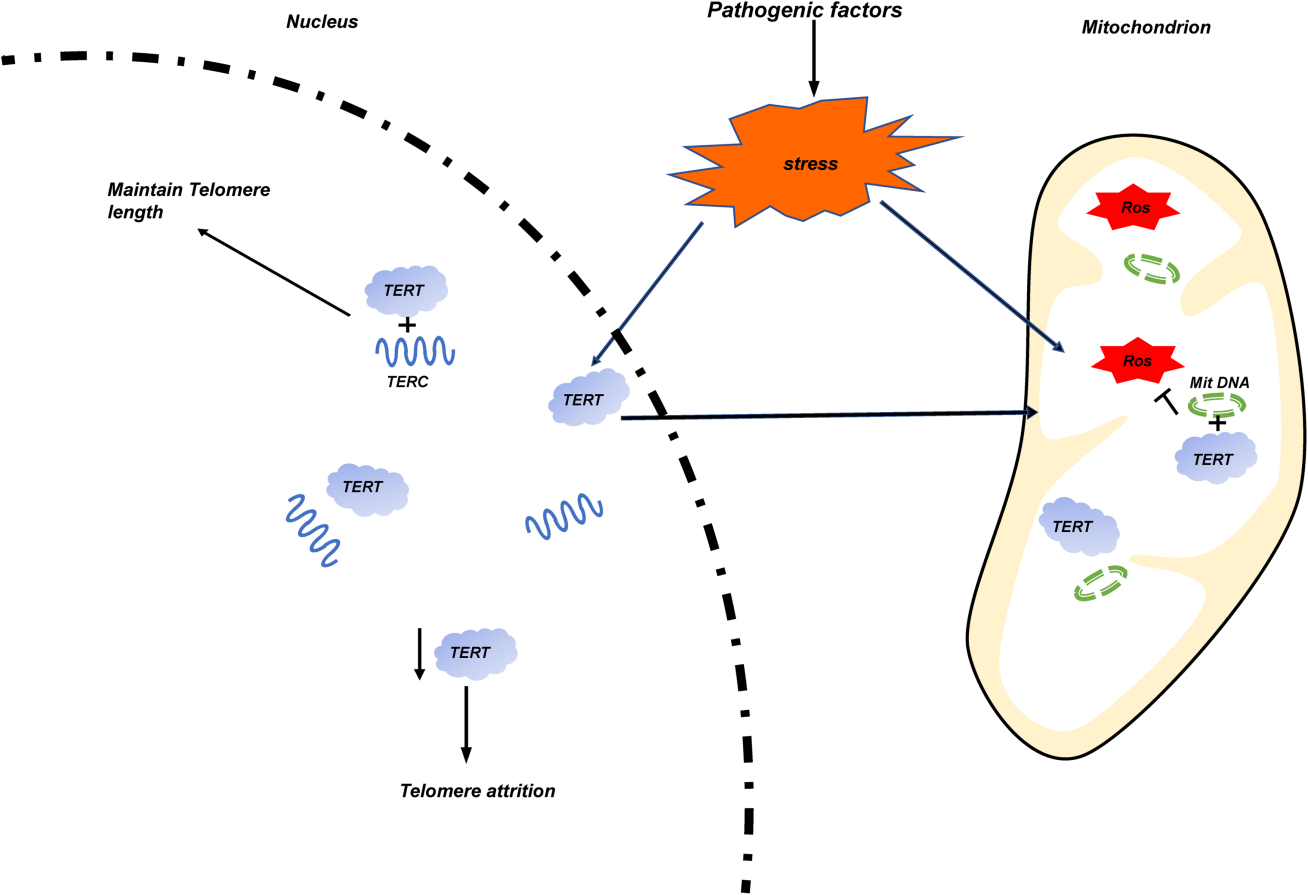
The occurrence of stress events is caused by pathogenic factors of various diseases. Mitochondrial damage increases the release of ROS, and TERT transfers from the nucleus to the mitochondria and binds with its circular DNA (Mit DNA) to inhibit the production of ROS. The reduction of the number of TERT in the nucleus leads to the wear of telomere length.

Telomere dysfunction is often associated with some adult cancers and aging related diseases. Since increasing evidence show that telomere dysfunction is related to the occurrence, development, and prognosis of some pediatric diseases, we systematically analyzed the relationship between telomere biology and some pediatric congenital and growth-related diseases in this article. Similar to that of adults, shorter TL is closely related to the pathological process of some children's diseases. To the preterm infants, shorter TL is mainly related to BPD and BA; For older children, studies on telomere dysfunction mainly focus on the following kinds of diseases: In endocrine system diseases, the influence of shorter TL on obese children of different genders is controversial. It is clear that the shorter TL is closely related to children's T1D in terms of disease occurrence and all-cause mortality of diseases; For Hereditary diseases, children with DS have longer telomeres, at the same time, they have higher telomere decay rate. For children with DC and DMD, telomere dysfunction is mainly related to the abnormality of telomere RNA structure TERC; Similar to the adult cancer, the overexpression and activation of TERT were also detected in most pediatric cancer patients such as NB, Brain tumor, et al. but there are exceptions, such as AML. There are also sporadic reports that telomere dysfunction is related to other body systems, such as cardiovascular system, respiratory system, digestive system and urinary system. Most of them are related to shorter TL. An interesting hypothesis is as follows. TERT acts as an RNA-dependent reverse transcriptase in the nucleus under stable conditions. When stress events occur, ROS production in mitochondria increases. At this time, TERT will translocated to mitochondria and combine with mitochondrial circular DNA (Mit DNA) to inhibit ROS production. The relative number of TERT in the nucleus decreases, resulting in telomere attrition. (Figure 4) Since telomere length is hereditary, it will be interesting to evaluate the correlation between the telomere length of newborns with diseases and the telomere length of their parents. This is of great significance for the long-term complications and treatment of newborns.

The length of telomeres at birth has a very important impact on the length of telomeres in children and adults. In the study of Martens DS et al. (117), the telomere length of newborns has a high predictive value to the telomere length of their later years. In the study of Martens DS et al. (118), it was found that the blood pressure of early school-age children was related to the length of telomere at birth. However, telomere length in childhood is also affected by other factors: Exposure to environmental pollution in early life is closely related to telomere wear (104, 119). Socioeconomic factor, and health and lifestyle factors also related to telomere wear (120). Sleep duration may also have a significant impact on TL in the first few years of life (121).

Most of the current treatments considering telomeres are based on the treatment of diseases related to adult telomere dysfunction. These therapeutic targets mainly focus on the intervention of TERT activity. Danazol, and BCH001 TA-65 used for endogenous TERT expression. AAV9-CMV-Tert/ AAV9-Tert used for exogenous import TERT. 1,3,4-Oxadiazole, GRN163L used for TERT inhibitor. Antisense strand RNA interference can also be used to inhibit TERT activity. The above treatment suggestions are based on the summary of the treatment of adult diseases and the validation of animal models. If they are applied to children's diseases, a large number of experiments are needed to verify their safety.

But telomerase activity and telomere length maintenance may be potential therapeutic targets for pediatric diseases.

## Author contributions

JF conceived the review and revised the manuscript. BZ performed the search, collect the data, and took charge of writing the original manuscript. All authors contributed to the article and approved the submitted version.
